# Personality and Lockdown: A Study on Italian Undergraduates During the COVID-19 Pandemic

**DOI:** 10.3389/fpsyt.2021.622366

**Published:** 2021-05-28

**Authors:** Silvia Biondi, Simona Casale, Jessica Burrai, Cristina Mazza, Gabriele Cavaggioni, Stefano Ferracuti, Anna Maria Giannini, Paolo Roma

**Affiliations:** ^1^Department of Human Neuroscience, Sapienza University of Rome, Rome, Italy; ^2^Department of Dynamic and Clinical Psychology and Health Studies, Sapienza University of Rome, Rome, Italy; ^3^Department of Psychology, Sapienza University of Rome, Rome, Italy; ^4^Department of Neuroscience, Imaging and Clinical Sciences, University G. d'Annunzio of Chieti Pescara, Chieti, Italy

**Keywords:** depression, anxiety, stress, compliance, defense mechanisms, prevention

## Abstract

The current study aimed at increasing our understanding of the psychological impact of the COVID-19 lockdown on undergraduate students, particularly with respect to the association between personality traits; defense mechanisms (DMs); depression, anxiety, and stress symptoms (DASSs); and compliance with the government recommended health measures. A sample of 1,427 Italian undergraduate students were administered the Personality Inventory for the DSM-5—Brief Form; the Defense Style Questionnaire-40; and the Depression, Anxiety and Stress Scale-21. Compliance with the COVID-19 behavioral recommendations was measured through a 10-item survey measure. Results showed that immature DMs and internalizing personality traits (i.e., detachment, negative affect, psychoticism) were risk factors of DASSs. Furthermore, subjects with higher levels of DASSs appeared less compliant with the health measures recommended by the Italian government. Experts may use these results to identify and subsequently support (*via* the Internet) young subjects at greater risk of mental health problems as a result of the COVID-19 pandemic.

## Introduction

Beginning in late 2019, the SARS-CoV-2 (i.e., COVID-19) virus spread extremely quickly around the world, resulting in the World Health Organization (WHO) declaring it a pandemic on 11 March 2020. Scientific reports have consistently indicated that quarantine measures to control the spread of COVID-19 are likely to trigger or exacerbate mental health problems, highlighting the need for a global response to reduce these negative consequences, in both pre-existing patients and the general population (1). Torales et al. (1) explained that COVID-19 has led to health problems in the general public, such as stress, anxiety, and depressive symptoms, as well as insomnia, denial, anger, and fear. Similarly, Mucci et al. (2) hypothesized, from a long-term perspective, that the COVID-19 pandemic would lead to increased instances of acute stress disorder (ASD), post-traumatic stress disorder (PTSD), emotional disturbance, sleep disorders, depressive syndromes, and suicides.

Studies have also aimed at identifying the risk and protective factors for psychological distress during the pandemic [e.g., (3–5)]. Mazza et al. (5) administered an online survey to 2,766 Italian participants (*M*_*age*_ = 32.94; *SD* = 13.2) from 18–22 March 2020. The survey included sociodemographic questions (i.e., age, gender, education), as well as the Depression, Anxiety and Stress Scale −21 item [DASS-21; (6)] and the Personality Inventory for DSM-5–Brief Form–Adult [PID-5-BF; (7)]. The results showed that female gender, negative affect, and detachment were associated with higher levels of depression, anxiety, and stress. Flesia et al. (4) assessed the stressful impact of the COVID-19 pandemic in Italy on 2,053 participants (*M*_*age*_ = 35.81), using an *ad hoc* online questionnaire to investigate participants' sociodemographic variables, health conditions, and personal history with COVID-19. Furthermore, they also administered the 10-item Perceived Stress Scale [PSS-10; (8)], the Coping Orientations to the Problems Experienced [COPENVI-25; (9)] measure, and the 10-item Big Five Inventory [BFI-10; (10)]. The results showed that participants with high agreeableness, high conscientiousness, high emotional stability, and high extraversion had lower levels of psychological distress. The authors also found that those with higher levels of perceived stress were less likely to adhere to the government's rules.

Considering the extremely critical period that led to the lockdown in Italy, and the ongoing global emergency—with repercussions for both physical and mental health—it is necessary to identify the people at greatest risk of suffering from the pandemic. To this end, several studies have analyzed specific target groups during the COVID-19 emergency, with the aim of providing indications for prevention and intervention programs in the event of a future outbreak (11–13). One such study, conducted by Fontanesi et al. (11), studied the effects of the lockdown on 1,126 Italian parents. Their findings suggested that parents of children diagnosed with a mental or physical disease experienced higher levels of parental burnout. These parents noted significant modifications in their children's behavior during the lockdown, and responded by shifting from an authoritative to an authoritarian parenting style, thus increasing their verbal hostility and decreasing their regulation reasoning. Hao et al. (14), instead, studied the psychological impact of the COVID-19 pandemic in China on psychiatric patients and healthy controls. They underlined a significant difference between groups in post-traumatic stress disorder (PTSD), depression, anxiety, stress, and insomnia, with psychiatric patients demonstrating a higher prevalence across all of these variables.

Undergraduate students comprise another vulnerable target group. The COVID-19 emergency has significantly impacted this population, primarily by limiting their contact with others. Students have been forced to drastically change their social lives, reduce their contact with peers, give up their hobbies, and replace their normal schooling with virtual education. Suddenly, they have had to prepare for exams without the help of their professors or friends; they have had lessons without being able to exchange their opinions; they have graduated in their own homes, together with only relatives; and they have been prevented from accessing traineeship programs, which are essential for their professional development. For these reasons, the challenges faced by this group are significant.

A group of Chinese researchers (15) evaluated the psychological condition of college students (*N* = 7,143) during the COVID-19 outbreak in China, administering the seven-item Generalized Anxiety Disorder Scale [GAD-7; (16)] and a set of basic questions (about, e.g., demographics, gender, and place of residence). The findings indicated that 24.9% of students were afflicted with anxiety. Furthermore, Gallè et al. (17) explored the link between behavior and knowledge about the COVID-19 pandemic in 2,125 (*M*_*age*_ = 22.5; *SD* = 0.08) Italian undergraduates at the Universities of Rome, Naples, and Bari. The results showed a good level of knowledge, with healthier behaviors indicated by females. However, in general, students did not modify their diet and smoking habits during the lockdown, and they decreased their physical activity.

The current study aimed at improving our understanding of Italian university students' psychological condition during the COVID-19 lockdown. In more detail, we sought to uncover whether this specific sample could be classified on the basis of personality variables (i.e., defense mechanisms and personality traits). Furthermore, we aimed at identifying possible differences between the emergent student groups in relation to depression, anxiety, stress symptoms (DASSs), and compliance with the recommended health measures (i.e., social distancing, wearing face masks, disinfecting hands).

## Materials and Methods

### Participants

Participants were 1,427 Italian undergraduate students (1,054 female, 373 male) from all the Faculties of the leading Italian universities of Padua, Florence, Rome, and Bari. Ages ranged from 18–27 years, with an average age of 23.26 years (*SD* = 2.27); this mean age is aligned with that of all Italian undergraduates, according to ISTAT data (2019).^1^ Participants were recruited online, they voluntarily and anonymously responded to the survey, which they accessed *via* a designated link, and they have not received any form of remuneration. Participants indicated informed consent prior to beginning the survey, and they were free to interrupt or quit the survey at any point without explanation. Data were collected over 1 week, from 1–7 April 2020.

Expedited ethics approval was obtained from the Institutional Board of the Department of Human Neuroscience, Faculty of Medicine and Dentistry, “Sapienza” University of Rome (IRB-2020-6), in conformity with the principles embodied in the Declaration of Helsinki.

### Materials

#### Personality Variables

Participants were administered the **Personality Inventory for the DSM-5–Brief Form** [PID-5-BF; (7, 18, 19)], which is an abbreviated version of the Personality Inventory for the DSM-5 [PID-5; (20)], designed to screen for dimensional maladaptive personality traits. The PID-5-BF–Adult is a 25-item self-rated personality trait assessment for adults aged 18 years and older. It measures five personality trait domains: negative affect, detachment, antagonism, disinhibition, and psychoticism. Each trait domain is measured by five items, with each item rated on a four-point Likert scale. Scores for the overall measure range from 0–75, with higher scores indicating greater overall personality dysfunction. Similarly, each trait domain score ranges from 0–15, with higher scores indicating greater dysfunction in the respective personality trait domain. The PID-5-BF has been validated in many countries, including Italy (21). The mean coefficient alpha ranges from 0.56–0.74, with a mean of 0.66 (21). Moderate to large correlations have been found between PID-5-BF domain subscales and their full-length PID-5 counterparts (22). In the present sample, the total test showed good internal consistency, with Cronbach's alpha of 0.85. Results were interpreted in relation to percentiles of the general Italian population (19).

To investigate participants' defense mechanisms, we administered the **Defense Style Questionnaire-40** [DSQ-40; (23)]. The DSQ-40 is a self-report instrument comprised of 40 items; thus, it is a shorter version of the Defense Style Questionnaire [DSQ; (24, 25)]. The scale measures respondents' defensive functioning *via* 20 defense mechanisms, categorized into three defense styles: mature, neurotic, and immature. The mature style includes sublimation, humor, anticipation, and suppression; the neurotic style includes undoing, pseudoaltruism, idealization, and reaction formation; and the immature style includes projection, passive aggression, acting out, isolation, devaluation, autistic fantasy, denial, displacement, dissociation, splitting, rationalization, and somatization. Each item is rated on a nine-point Likert scale extending from 1 (*strongly disagree*) to 9 (*strongly agree*). The DSQ-40 has been validated in many countries, including Italy, and has shown sufficient internal consistency, with α = 0.61, 0.59, and 0.80 for mature, neurotic, and immature defense styles, respectively (23). In the present sample, Cronbach's alphas for the mature, neurotic, and immature defense styles were, α = 0.62, 0.62, and 0.68, respectively.

#### Actuarial Variables

To investigate participants' levels of depression, anxiety, and stress, we administered the **Depression, Anxiety and Stress Scale-21** [DASS-21; (6)]. The DASS-21 is a short form of the 42-item self-report DASS measure. The three DASS-21 subscales measure depression, anxiety, and stress, respectively. Each subscale is comprised of seven items, which are scored on a four-point Likert scale ranging from 0 (*does not apply to me at all*) to 3 (*applies to me very much, or most of the time*). Higher scores indicate more frequent symptomatology. The DASS-21 has been validated in many countries, including Italy, and has been found to show good internal consistency for the Depression, Anxiety, and Stress subscales, with α = 0.82, 0.74, and 0.85, respectively (26). In the present sample, Cronbach's alphas for the Depression, Anxiety and Stress subscales were α = 0.88, 0.83, and 0.91, respectively. Cronbach's alpha for the total scale was 0.94.

**Compliance** with the government's recommended health measures to control the spread of COVID-19 was measured *via* 10 questions (e.g., “It is suggested that all persons avoid crowed places. Are you complying with this?”). Each question was assessed on a five-point Likert scale ranging from 1 (*hardly*) to 5 (*extremely*). The survey showed good internal consistency, with Cronbach's alpha of 0.82.

### Data Analysis

A two-step cluster analysis with the AIC and the BIC criteria was used to define the profile of Italian undergraduate students. The two-step cluster analysis is an explanatory tool designed to: (a) reveal natural groupings within a data set without modifying the data, and (b) identify homogenous subgroups presenting similar characteristics. In the present analysis, the cluster model incorporated the investigated personality traits (as measured by the PID-5-BF) and defense mechanisms (as measured by the DSQ-40). To achieve natural clustering, the number of clusters was set to automatic.

Multivariate and univariate analyses of variance (MANOVAs and ANOVAs) were run to identify differences between the emergent three clusters pertaining to depression, anxiety, and stress levels (as assessed by the DASS-21) and compliance with the recommended health behaviors. Statistical analyses were conducted using the software package SPSS, version 25.

## Results

### Cluster Analysis

The sample-size adjusted and the likelihood distances of both the AIC and the BIC criterion for the two-step cluster analysis are reported in Table 1.

**Table 1:** Sample-size adjusted and likelihood distances of the AIC and the BIC criteria.

	**AIC criterion**			**BIC criterion**		
**Number of Clusters**	**AIC Changes^a^**	**Ratio of AIC Changes^b^**	**Ratio of Distance Measures^c^**	**BIC Changes^a^**	**Ratio of BIC Changes^b^**	**Ratio of Distance Measures^c^**
1						
2	−1351.438	1.000	2.182	−1267.225	1.000	2.182
3	−601.896	0.445	1.995	−517.683	0.409	1.995
4	−285.724	0.211	1.577	−201.511	0.159	1.577
5	−169.534	0.125	1.051	−85.321	0.067	1.051
6	−159.685	0.118	1.040	−75.472	0.060	1.040
7	−152.259	0.113	1.038	−68.045	0.054	1.038
8	−145.586	0.108	1.254	−61.373	0.048	1.254
9	−109.634	0.081	1.106	−25.420	0.020	1.106
10	−96.096	0.071	1.191	−11.883	0.009	1.191
11	−75.564	0.056	1.251	8.649	−0.007	1.251
12	−53.969	0.040	1.197	30.245	−0.025	1.197
13	−39.807	0.029	1.109	44.406	−0.035	1.109
14	−32.733	0.024	1.078	51.480	−0.041	1.078
15	−28.039	0.021	1.079	56.174	−0.044	1.079

a *The changes are from the previous number of clusters in the table*.

b *The ratios of changes are relative to the change for the two cluster solution*.

c *The ratios of distance measures are based on the current number of clusters against the previous number of clusters*.

The two-step cluster analysis of the 1,427 participants revealed three clusters with significant differences in mean score profiles (see Table 2). Characteristics of each cluster were as follows:

• Cluster 1 was composed of subjects with a tendency to develop dysfunctional levels of psychoticism, negative affect, and antagonism (at about the 75th percentile), as measured by the PID-5-BF. However, subjects in this group had average scores on all three DSQ-40 defense style subscales. This was the largest cluster, representing 38.6% of the sample.
• Cluster 2 was composed of subjects whose scores on the PID-5-BF revealed dysfunction in negative affect, psychoticism, and detachment (at about the 95th percentile), and who registered higher than average scores on the Neurotic and Immature subscales of the DSQ-40. This cluster was the smallest, representing 27.3% of the sample.
• Cluster 3 was composed of subjects who showed no dysfunction in any PID-5-BF domain and registered average scores on all three DSQ-40 subscales. This cluster represented 34.1% of the total sample.

**Table 2:** Two-step cluste analysis.

	**Cluster 1 *N* = 551 (38.6%) M (*SD*)**	**Cluster 2*N* = 390 (27.3%)M (*SD*)**	**Cluster 3 *N* = 486 (34.1%) M (*SD*)**
**PID-5-BF**			
Psychoticism	0.93 (0.40)	1.57 (0.46)	0.43 (0.31)
Detachment	0.72 (0.39)	1.37 (0.46)	0.28 (0.027)
Negative Affect	1.29 (0.44)	1.79 (0.41)	0.78 (0.47)
Disinhibition	0.83 (0.52)	1.25 (0.62)	0.36 (0.37)
Antagonism	0.69 (0.47)	0.94 (0.52)	0.27 (0.26)
**DSQ-40**			
Immature DM	4.14 (0.77)	5.12 (0.89)	3.23 (0.74)
Neurotic DM	5.35 (1.50)	6.25 (1.37)	5.06 (1.48)
Mature DM	4.97 (1.26)	5.08 (1.35)	4.95 (1.31)

**Figure 1:**
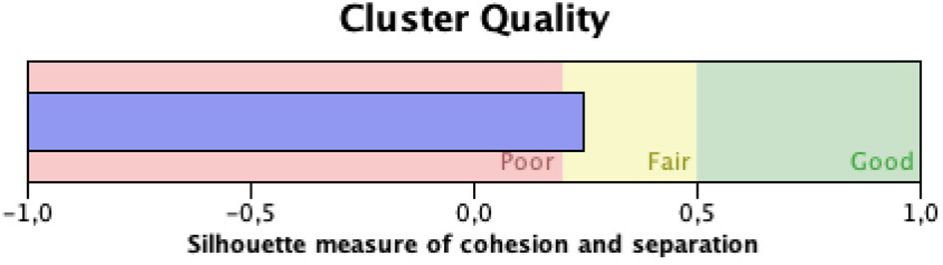
Quality of the two-step cluster analysis.

Information about silhouette measure of cohesion and separation of the Cluster Analysis is reported in Figure 1.

### Between Group Comparison (MANOVA and ANOVAs)

A 3 × 3 MANOVA showed a significant clustering effect between the DASS-21 subscales [*V* = 0.177, *F*_(6, 2846)_ = 46.151, *p* < 0.001, *par*η^2^ = 0.089]. In more detail, separate univariate ANOVAs on the outcome variables revealed a significant clustering effect on all DASS-21 subscales (see Table 3). One ANOVA also showed a significant clustering effect between Cluster 2 and Clusters 1 and 3 on compliance with the recommended health measures.

**Table 3:** Between group comparison (MANOVA and ANOVAs).

	**Cluster 1 *N* = 551 (38.6%)**	**Cluster 2*N* = 390 (27.3%)**	**Cluster 3 *N* = 486 (34.1%)**	***F***	***p***	**parη^2^**
DASS-21						
Depression	6.09 (4.74)^a^	9.13 (5.53)^b^	3.79 (3.60)^c^	144.22	<0.001	0.168
Anxiety	3.07 (3.67)^a^	5.07 (4.53)^b^	2.10 (3.07)^c^	69.31	<0.001	0.089
Stress	8.25 (5.41)^a^	10.76 (5.88)^b^	6.00 (4.69)^c^	86.87	<0.001	0.109
Compliance	41.68 (5.71)^a^	40.46 (6.30)^b^	42.52 (5.61)^a^	13.51	<0.001	0.019

*For each line, different letters indicate a significant difference between columns*.

Cluster 2 subjects showed the highest average scores on all DASS-21 subscales, but particularly the Stress subscale. Cluster 3 subjects showed the lowest average scores on all DASS-21 subscales, especially the Anxiety subscale; their highest score (within group comparison) was on the Stress subscale. An ANOVA showed a significant difference between Cluster 2 and the other two Clusters on compliance with the recommended health measures (*F* = 13.51, *p* < 0.001, *par*η^2^= 0.019), with Cluster 2 scoring lower than the other groups.

Following, a list of the 10 behaviors analyzed to compute subject's compliance with the health-related measures imposed by the Italian Government (see Table 4).

**Table 4:** Between group comparison (MANOVA).

	**Cluster 1 *N* = 551 (38.6%)**	**Cluster 2*N* = 390 (27.3%)**	**Cluster 3 *N* = 486 (34.1%)**	***F***	***p***	**parη^2^**
**Compliance**						
Avoid hugs	3.78 (1.22)^a^	3.61 (1.28)^a^	3.78 (1.20)^a^	2.718	0.066	0.004
Avoid hands shake	4.52 (0.77)^a^	4.36 (0.89)^b^	4.55 (0.75)^a^	6.586	0.001	0.009
(At least) 1 meter distance	3.94 (1.04)^a, b^	3.83 (1.11)^a^	4.10 (0.94)^b^	8.024	<0.001	0.011
Avoid drinking from bottles and glasses used by others	4.55 (0.78)^a^	4.38 (0.92)^b^	4.61 (0.72)^a^	9.415	<0.001	0.013
Avoid crowed places	4.63 (0.68)^a, b^	4.54 (0.79)^a^	4.69 (0.59)^b^	5.183	0.006	0.007
Frequent handwashing at home	4.13 (1.02)^a^	4.06 (1.06)^a^	4.20 (0.91)^a^	2.319	0.099	0.003
Frequent handwashing outside	4.35 (0.94)^a, b^	4.28 (0.97)^a^	4.45 (0.82)^b^	3.951	0.019	0.006
Avoid touching your face	3.05 (1.23)^a^	2.92 (1.25)^a^	3.27 (1.18)^b^	9.350	<0.001	0.013
Sneeze and cough into a handkerchief or in the elbow	4.20 (0.96)^a, b^	4.12 (0.98)^a^	4.32 (0.89)^b^	5.188	0.006	0.007
Stay at home	4.53 (0.74)^a^	4.36 (0.88)^b^	4.55 (0.72)^a^	7.531	0.001	0.010

*For each line, different letters indicate a significant difference between columns*.

A 3 × 10 MANOVA showed a significant clustering effect between the 10 compliant behaviors [*V* = 0.031, *F*_(2, 1424)_ = 2.219, *p* = 0.001, *par*η^2^ = 0.015]. In more detail, separate univariate ANOVAs on the outcome variables revealed a significant clustering effect on “Avoid hands shake,” “Avoid drinking from bottles and glasses used by others,” and “Stay at home” between Cluster 2 and the other two Clusters, with Cluster 2 showing lower scores; on “(At least) 1 meter distance,” “Avoid crowed places,” “Frequent handwashing outside,” and “Sneeze and cough into a handkerchief or in the elbow” between Cluster 2 and Cluster 3, with Cluster 2 scoring lower; on “Avoid touching your face” between Cluster 3 and the other two Clusters, with Cluster 3 showing higher scores.

## Discussion

The main goal of the present study was to better understand the condition of Italian undergraduate university students during the COVID-19 lockdown, considering the important repercussions that this extraordinary measure had on their lives. Most notably, the lockdown significantly changed the ways in which they attended classes, prepared for and dealt with exams, earned degrees, and, more generally, navigated their social lives.

To answer to our research question, we first classified the sample of students according to personality traits and defense mechanisms. Three clusters of students emerged, grouped by common personality traits and defense mechanisms. Cluster 1 subjects showed a slight tendency to display dysfunction in some personality areas, such as negative affect, psychoticism, and detachment, as measured by the PID-5-BF. Therefore, these participants may have experienced negative emotions—with possible non-adaptive behavioral and interpersonal consequences—, tried to avoid socioemotional interactions, and had limited ability to feel pleasure (21). On the other hand, they did not show compromised defense mechanisms or dysfunctional emotional and perceptive processing. Cluster 2 subjects revealed dysfunctional traits in the PID-5-BF areas of negative affect, psychoticism, and detachment, with important behavioral and relational impairment, negative emotions (e.g., worry, anger, anxiety, and depression), withdrawal from interpersonal interactions, and inconsistent thought processes and contents (21). They also showed a suppression of emotional conflict and a distorted awareness of unpleasant psychological events (27). Finally, Cluster 3 subjects showed overall personality functioning, with no significantly compromised behavioral or interpersonal aspects or defense styles.

As a second step, we investigated whether depression, anxiety, and stress levels differed between the identified clusters. The findings showed significant differences with respect to all of the aforementioned psychological variables, with Cluster 2 subjects showing the worst performance. Thus, although the results highlighted higher mean scores for the DASS-21 subscales in all three clusters than those indicated by Bottesi et al. (26)—with the exception of the mean Anxiety subscale score for Cluster 3—, students in Cluster 2 still experienced DASSs during the lockdown and presented the highest scores on the DASS-21 Stress subscale. Finally, students in Cluster 2 also demonstrated less compliance with the recommended health measures imposed by the Italian government, compared to Clusters 1 and 3. In fact, Cluster 2 subjects reported less adherence to recommended measures, showing a tendency to avoid shaking hands and drinking from others' bottles and glasses less than their peers, as well as staying less at home. Although significant, the difference between Clusters on Compliance resulted smaller than the one on all DASS-21 subscales. It is worth noting that Cluster 2 demonstrated the worst performance on all personality variables (as measured by the PID-5-BF and DSQ-40); thus, we might hypothesize that students with worse personological functioning were most affected by the COVID-19 lockdown, with repercussions for their psychological well-being and compliance with the recommended health measures.

The present findings are consistent with those obtained in previous studies (4, 5). In particular, avoidance of socioemotional interactions and limited ability to feel pleasure (as measured by the PID-5-BF Detachment subscale) have previously been found to be correlated with higher distress (5), and negative emotions such as worry, anger, anxiety, and depression (as measured by the PID-5-BF Negative Affect subscale) have been found to have a greater effect on stress caused by an epidemic (4). Thus, even in the present study, negative emotionality seemed to have an effect on the stress helicitated by the COVID-19 pandemic, confirming the results previously obtained. With respect to the PID-5-BF Psychoticism subscale, which emerged as the best predictor in the cluster model, numerous studies [e.g., (22)] have found this subscale to have low discriminating validity and to be moderately correlated with other PID-5-BF domains. Furthermore, it has been found to be a good predictor of depression levels (22).

Concerning the DSQ-40, Cluster 2 students scored higher on the Immature Defense subscale, relative to students in Clusters 1 and 3. This result is aligned with the findings of Granieri et al. (28), who examined the relationship between DMs and personality traits in an adult sample aged 18–64 years, using the DSQ-40 and PID-5-BF. The authors found that DMs were significant predictors of PID-5-BF total scores. In particular, scores on the Immature subscale predicted higher scores on the PID-5-F maladaptive personality subscales; and scores on the Mature subscale negatively predicted total PID-5-BF scores.

The present results support the idea of a close link between DMs and personality functioning. Thus, it may be useful for experts to assess young people using both the PID-5-BF and the DSQ-40, in order to obtain an accurate screening. In particular, the use of these two short tests, administered *via* the Internet, could help experts quickly identify subjects at greater risk for psychological distress and lower compliance with the recommended health measures, and enable them to implement a valid and *ad hoc* intervention strategy.

Overall, the present results highlight that Italian undergraduate students experienced DASSs during the COVID-19 lockdown, with stress symptoms the most pronounced—in accordance with the Chinese data (15). These findings represent an opportunity to reflect on the connection between mental health and restrictive measures in the context of an epidemic, highlighting the urgent need to define a strategy to identify subjects at greater risk of developing psychopathology in this situation. This is particularly important with respect to the population examined here, given that young people tend to frequent crowded places, and if they do not comply with the health measures recommended by the government, they risk spreading COVID-19.

## Strengths and Limitations

It should be borne in mind that the lockdown in Italy was unique, because Italy was the first country in Europe to have a significant number of deaths from COVID-19 and to adopt strong restrictions to curb the spread of the virus. Italian citizens were thus caught on the hop and not prepared for such drastic changes to their lifestyles; this may have had a strong influence on their mental health. For this reason, while our results may provide important information regarding the psychological reaction of undergraduate students to the COVID-19 situation in Italy, the findings are not necessarily generalizable to students in other countries.

The impact of the COVID-19 emergency on peoples' lives, attitudes, and behaviors is ongoing. For instance, in their attempts to avoid crowds, Italians—and particularly younger Italians—are opting to engage more online. Thus, by administering preventive measures *via* the Internet, experts may reach a higher number of people at risk for mental disease in less time, thereby avoiding the spread of psychopathologies in the coming years and promoting compliance with the government-recommended health measures (29).

The present study also has certain limitations, and should be interpreted with caution. First, the study was implemented during the first phase of the COVID-19 outbreak in Italy, and we were unable to assess students' psychological functioning before the lockdown, as well as their compliance with the recommended health measures in more advanced phases of the outbreak. Thus, we were prevented from drawing causal inferences. Furthermore, the present study assessed only undergraduate students, and excluded high school, middle school, and primary school students. Finally, our sample was mostly comprised of mostly females, so further studies should reproduce our research with other Italian students, including more male participants.

## Conclusion

The present results highlight the significant impact of the COVID-19 lockdown in Italy on the mental health of undergraduate students, showing a common experience of depression, anxiety, and stress—possibly dependent on personality functioning. Students with significant behavioral and relational impairments (i.e., those who experienced negative emotions and withdrew from interpersonal interactions) and compromised defense styles were more at risk of developing DASSs and not complying with the health measures recommended by the Italian government.

Furthermore, the results suggest that a preliminary assessment with two short tests (PID-5-BF and DSQ-40) might effectively identify people at greater risk of developing mental health problems and those who are less likely to comply with the rules set out by the government; such an assessment might also inspire a sense of civic duty in respondents, with positive effect.

We hope that these findings offer valuable insight into the situation of Italian undergraduate students following the COVID-19 lockdown and indicate a possible approach with this specific population, in the event of a future emergency situation.

## Data Availability Statement

The raw data supporting the conclusions of this article will be made available by the authors, without undue reservation.

## Ethics Statement

The studies involving human participants were reviewed and approved by Board of the Department of Human Neuroscience, Faculty of Medicine and Dentistry, Sapienza University of Rome (IRB-2020-6). The patients/participants provided their written informed consent to participate in this study.

## Author Contributions

SB, SC, and JB: online questionnaire implementation. CM: data analysis. PR, SB, JB, CM, and SF: data interpretation. SB, JB, CM, SC, GC and AG: drafting of manuscript. All authors: survey conception, revised the manuscript critically and gave final approval of the version to be published.

## Conflict of Interest

The authors declare that the research was conducted in the absence of any commercial or financial relationships that could be construed as a potential conflict of interest.

## Footnotes

^1^http://dati-giovani.istat.it/Index.aspx#
